# Effects of Recycled Fe_2_O_3_ Nanofiller on the Structural, Thermal, Mechanical, Dielectric, and Magnetic Properties of PTFE Matrix

**DOI:** 10.3390/polym13142332

**Published:** 2021-07-16

**Authors:** Ahmad Mamoun Khamis, Zulkifly Abbas, Raba’ah Syahidah Azis, Ebenezer Ekow Mensah, Ibrahim Abubakar Alhaji

**Affiliations:** 1 Department of Physics, Faculty of Science, Universiti Putra Malaysia, Serdang 43400, Selangor, Malaysia; akhameis@yahoo.com (A.M.K.); rabaah@upm.edu.my (R.S.A.); alhaji259@gmail.com (I.A.A.); 2 Introp, University Putra Malaysia, Serdang 43400, Selangor, Malaysia; 3 Institute of Advanced Material, University Putra Malaysia, Serdang 43400, Selangor, Malaysia; 4 Faculty of Science Education, University of Education, Winneba, P.O. Box 40 Mampong, Ashanti, Ghana; ebenekowmensah@aamusted.edu.gh

**Keywords:** PTFE, nanoparticles, microwave, recycled Fe_2_O_3_, complex permittivity, complex permeability

## Abstract

The purpose of this study was to improve the dielectric, magnetic, and thermal properties of polytetrafluoroethylene (PTFE) composites using recycled Fe_2_O_3_ (rFe_2_O_3_) nanofiller. Hematite (Fe_2_O_3_) was recycled from mill scale waste and the particle size was reduced to 11.3 nm after 6 h of high-energy ball milling. Different compositions (5–25 wt %) of rFe_2_O_3_ nanoparticles were incorporated as a filler in the PTFE matrix through a hydraulic pressing and sintering method in order to fabricate rFe_2_O_3_–PTFE nanocomposites. The microstructure properties of rFe_2_O_3_ nanoparticles and the nanocomposites were characterized through X-ray diffraction (XRD), field emission scanning electron microscopy (FESEM), and high-resolution transmission electron microscopy (HRTEM). The thermal expansion coefficients (CTEs) of the PTFE matrix and nanocomposites were determined using a dilatometer apparatus. The complex permittivity and permeability were measured using rectangular waveguide connected to vector network analyzer (VNA) in the frequency range 8.2–12.4 GHz. The CTE of PTFE matrix decreased from $65.28\times 10^{-6}/{}^{\circ}C$ to $39.84\times 10^{-6}/{}^{\circ}C$ when the filler loading increased to 25 wt %. The real (*ε*′) and imaginary (*ε*″) parts of permittivity increased with the rFe_2_O_3_ loading and reached maximum values of 3.1 and 0.23 at 8 GHz when the filler loading was increased from 5 to 25 wt %. A maximum complex permeability of $\left(1.1-j0.07\right)$ was also achieved by 25 wt % nanocomposite at 10 GHz.

## 1. Introduction

Mill scale scrap is considered as a solid waste generated in the steel and iron industries. In general, nearly (8–15) kg of mill scale waste is produced per 1000 kg of steel, and the annual production of mill scale waste is over 13.5 million tons according to preliminary statistics. Moreover, most of this waste is stockpiled in factories due to its limited utilization. The iron protoxide in this waste can be dissolved in acid rain and subsequently transferred into soil and ground waters. Mill scale waste has a dual concern with respect to environmental pollution and land occupancy. Therefore, it is crucial to develop a cleaner method to solve the abundant mill scale resource issue [1].

The study of synthetic techniques for ferrite nanoparticles production has recently attracted considerable attention in view of their excellent magnetic and electrical properties [2]. Up to date, various techniques have been used for the synthesis of Fe_2_O_3_ nanoparticles (Reference code: 00-024-0072) such as electrospinning technique [3], reduction-oxidation process [4], and hydrolysis process [5]. Indeed, the latter techniques have many disadvantages such as: friability of inorganic nanofibers after calcination [6], non-cost-effective for high contaminant concentrations, and slow reaction speed, respectively. However, mill scale waste materials have never been used to obtain Fe_2_O_3_ (00-024-0072) yet.

The microwave absorbing materials (MAMs) are in high demand to absorb the unwanted electromagnetic waves via dielectric and magnetic losses [7]. Microwave materials are used in the frequency range (300 MHz–300 GHz) [8]. MAMs are extensively used for many military and commercial applications such as electromagnetic shielding [9]. Therefore, the development of polymer composites (PCs) with tunable dielectric and magnetic properties have triggered researchers in recent years. The PCs reinforced with nanofillers have been widely studied to provide improved radiation protection properties compared to their metal counterparts. In addition, the electronics, packaging, household, energy, sports, communication, and leisure industries are fully involved in the use of PCs for various applications [10]. The advanced polymer composites can be fabricated based on the requirement by selecting quantity, shape, size, and type, of fillers as the reinforcements in different polymer matrix [11].

Polytetrafluoroethylene (PTFE) is a thermoplastic polymer, and PTFE is the best solvent chemical with the best resistance among thermoplastics. The properties of PTFE such as high service temperature, low moisture absorption, chemical inertness, etc., are crucial for many microwave applications [12]. However, PTFE also has certain disadvantages such as high coefficient of thermal expansion (CTE) [13,14], and low relative complex permittivity (*ε*^∗^) [15]. In addition, several studies have used Fe_2_O_3_ as the filler in PTFE matrix. Madusanka et al. synthesized thick film gas sensor using PTFE and α-Fe_2_O_3_ nanoparticles [16] while Kang et al. fabricated porous Fe_2_O_3_-PTFE nanofiber membranes for photocatalysis applications [17]. Moreover, PTFE-Al-Fe_2_O_3_ composites were prepared to be used as reactive materials [18]. The electro-Fenton system was synthesized by combining multiwall carbon nanotubes and Fe@Fe_2_O_3_ nanowires with PTFE [19]. It is also worth noting that Fe_2_O_3_ nanoparticles have been widely used as fillers to enhance the dielectric and magnetic properties of various matrixes such as polycaprolactone (PCL) [2], polyaniline (PANI) [20], silica [21], and poly(methyl methacrylate) (PMMA) [22]. However, rFe_2_O_3_ nanoparticles have never been used as fillers in PTFE matrix for microwave absorption applications yet.

In this study, both CTE and *ε*^∗^ were improved by incorporating rFe_2_O_3_ into a PTFE matrix. The rFe_2_O_3_ nanofiller with enhanced relative complex permittivity can be embedded with a non-conducting polymer matrix such as PTFE to prepare a novel absorber which could have promising attenuation properties for electromagnetic interference (EMI) suppression. This absorber can be further enhanced by the magnetic nature of the Fe_2_O_3_ nanoparticles and with their high interfacial density due to their good compactness [23].

Knowledge of the complex permittivity and complex permeability of materials has sparked a great interest in industrial and scientific applications [24]. The interaction between a dielectric sample and high frequency electromagnetic signal can be expressed by the relative complex permittivity equation: $\varepsilon^{*}=\varepsilon^{\prime }-j\varepsilon^{\prime \prime },$ where *ε*′ and *ε*″, respectively, represent the real and imaginary parts. The ratio of $tan\delta =\varepsilon^{\prime \prime }/\varepsilon^{\prime }$ represents the loss tangent of a sample and higher values indicate higher attenuation properties [25]. In Addition, the interaction effect between a specimen and the magnetic field component of EM waves can be characterized by the relative complex permeability (µ^∗^) which can be expressed by the equation: ${µ}^{*}={µ}^{\prime }-j{µ}^{\prime \prime }$ where µ′ and µ″ represent the real and imaginary parts, respectively [26]. The real part determines the amount of energy that the material has stored from an external magnetic field, while the imaginary part measures the attenuation of the magnetic field by the material. The magnetic loss tangent ($tan\delta_{µ})\;$is calculated using the equation $tan\delta_{µ}=\frac{{µ}^{\prime \prime }}{{µ}^{\prime }}$ [27], which represents the power lost versus power stored in a sample [28].

In this research, a low-cost, less complicated and non-chemical synthesis of recycled ferrite was done from mill scale waste. Then, the particle size of rFe_2_O_3_ decreased to nano-size via high energy ball milling (HEBM) in order to enhance the dielectric properties. The current study also involves the processes of synthesizing five nanocomposites of rFe_2_O_3_—PTFE by varying the rFe_2_O_3_ percentage in the nanocomposites. Afterwards, the resultant effects of rFe_2_O_3_ loadings on the phase composition, thermal expansion tensile strength, density, complex permittivity, and complex permeability of PTFE matrix were investigated.

## 2. Materials and Methods

### 2.1. Materials

The materials used for the preparation of the rFe_2_O_3_ nanoparticles and rFe_2_O_3_–PTFE nanocomposites were: Mill scale flakes (Perwaja Sdn. Bhd., Chukai, Terengganu, Malaysia) and PTFE molding powder (Fujian Sannong New Materials Co., LTD, Sanming, China) with average particle size 50–110 μm.

### 2.2. Synthesis of rFe_2_O_3_ Nanopartilces from Mill Scale

**Figure 1:**
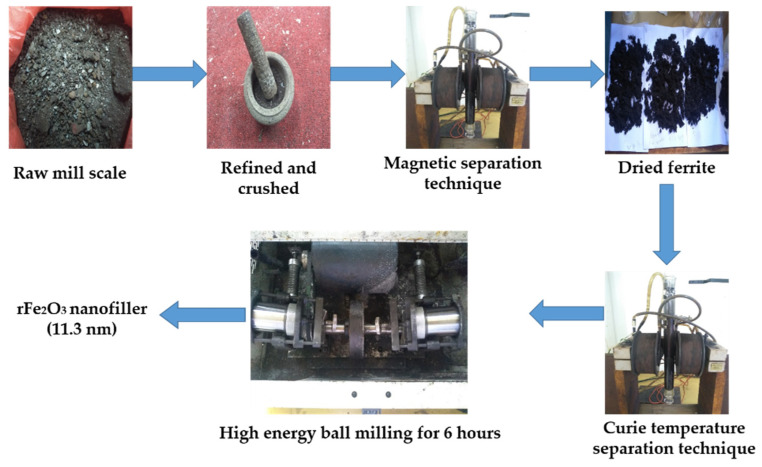
Preparation process of rFe_2_O_3_ nanoparticles.

Mill scale waste was initially cleaned by removing the impurities and then crushed manually into powder using mortar. This was followed by the application of the magnetic separation technique (MST) and Curie temperature separation technique (CTST) to further purify the powdered mill scale. The materials used for these processing techniques were an electromagnet which produced 1 Tesla magnetic field intensity, a thin cylindrical glass tube open at both ends and deionized water of density 1 g/cm^3^. The CTST was used to separate the wustite (FeO) and magnetite (Fe_3_O_4_) contained in the mill scale slurry collected in the cold water separation process [23]. In this separation step, the clamped glass tube closed at the bottom end with a glass stopper, was filled with hot deionized water (100 °C) followed by a small quantity of the slurry. The particles that remained attracted to the magnetic field were inferred to be magnetite (Fe_3_O_4_) because of its higher Curie temperature of 585 °C. The rFe_3_O_4_ powder obtained was milled via the mechanical alloying technique using the high energy ball mill (SPEX Sample Prep 8000D, Metuchen, NJ, USA) operated by a 1425 rpm 50 Hz motor at a clamp speed of 875 cycles/minute. The powder to balls ratio of 1:10 was used. A total of 7 gm of the Fe_3_O_4_ powder was poured into each of the two vials containing the 70 g of steel balls and milled for 6 h to produce nanoparticles. During the ball milling Fe_3_O_4_ was converted to Fe_2_O_3_ due to high temperature and the content of oxygen in the vials. The preparation process of rFe_2_O_3_ nanoparticles is shown in Figure 1.

### 2.3. Fabrication of rFe_2_O_3_–PTFE Nanocomposites

**Figure 2:**
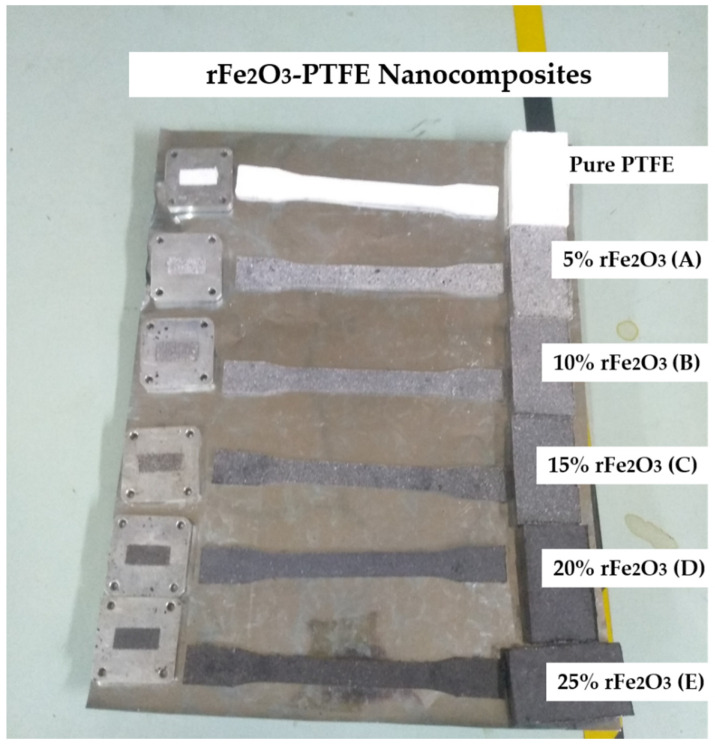
rFe_2_O_3_–PTFE nanocomposites.

The rFe_2_O_3_–PTFE nanocomposites shown in Figure 2 were fabricated by mixing PTFE powder with different mass percentages (5–25%) of the rFe_2_O_3_ nano-powder as listed in Table 1. The composites were prepared through a hydraulic pressing and sintering method.

**Figure 3:**
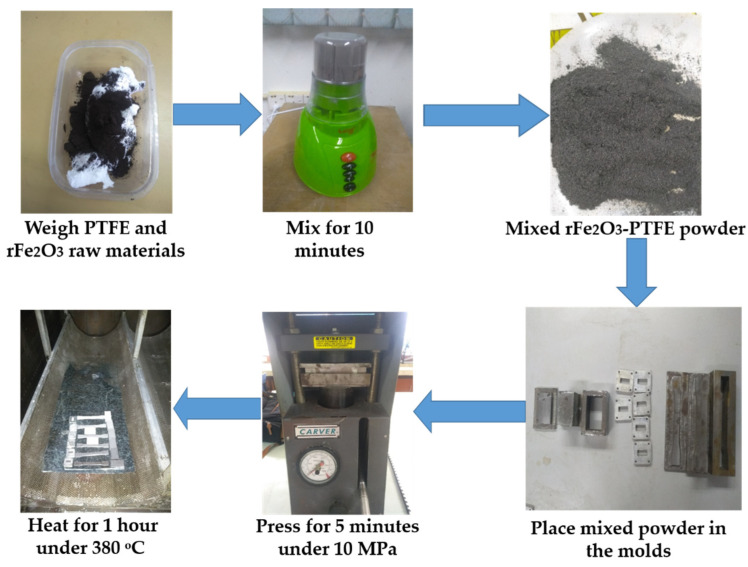
rFe_2_O_3_–PTFE nanocomposites preparation.

Three different shapes of nanocomposites were fabricated for each mass percentage in order to suite different characterization apparatuses. The raw materials were weighed using analytical micro balance (A&D Company, Ltd., GR-200, Tokyo, Japan) which has an accuracy of ±0.00007 g. The mixing was performed via Wing mixer/blender for 10 min. Then, the mixed powders were placed into three different molds and pressed using a hydraulic pressing machine for 5 min under 10 MPa load. Finally, the compact rFe_2_O_3_–PTFE nanocomposites were sintered as follows: the compact nanocomposites were heated from room temperature to 380 °C in a furnace at 2.97 °C/min and then maintained for 1 h to allow the PTFE particles to coalesce completely. The cooling process was 1 °C/min from 380 °C to room temperature. The rFe_2_O_3_–PTFE nanocomposites preparation is shown in Figure 3. After cooling, the rFe_2_O_3_–PTFE nanocomposites were ready for characterizations.

### 2.4. Characterization

rFe_2_O_3_ nanoparticles, PTFE powder, rFe_2_O_3_–PTFE nanocomposites were characterized using the following techniques:

#### 2.4.1. X-ray Diffraction (XRD)

The phase composition structure of the samples was analyzed using X-ray diffraction (XRD). The data were collected using a fully automated Philips X’pert system (Model PW3040/60 MPD, Amsterdam, The Netherlands) with Cu–Kα radiation operating at a current of 40 mA, a wavelength of 1.5405 Å, and a voltage of 40 kV. The diffraction patterns were recorded with a scanning speed of 2°/min in the 2Ѳ range 10 to 70°. The collected data were subjected to the Rietveld analysis on PANalytic X’Pert Highscore Plus v.3.0 software (PANalytical B.V., Almelo, The Netherlands). The diffraction peaks were compared with the Inorganic Crystal Structure Database (ICSD). The rFe_2_O_3_–PTFE samples were cut from rFe_2_O_3_–PTFE nanocomposites prepared in advanced in a solid form, while the rFe_2_O_3_ was in the form of a fine powder.

#### 2.4.2. High-Resolution Transmission Electron Microscopy (HRTEM)

The size and shape of rFe_2_O_3_ nanoparticles were investigated using HRTEM (JEM-2100F, JEOL, Tokyo, Japan). Drops of rFe_2_O_3_ nanoparticles were dispersed and placed on TEM grids for drying. The dried specimen was then placed in a high-vacuum chamber of the microscope for viewing and analysis of the nanoparticles.

#### 2.4.3. Field Emission Scanning Electron Microscopy (FESEM)

The dispersion of rFe_2_O_3_ nanoparticles in PTFE matrix was evaluated using FESEM (JEOL Ltd. JSM-7600, Tokyo, Japan). Carbon tapes were used to cover the aluminums stubs before placing the specimens on them. Then, the samples were coated with titanium using an auto-fine coater (JEOL Ltd. JEC-3000FC, Tokyo, Japan) to increase their conductivity and eliminate electromagnetic charges. Finally, the stubs were placed on the FESEM chamber for analysis.

#### 2.4.4. Electronic Densitometer

The effects of rFe_2_O_3_ nanoparticles on the density of PTFE matrix was determined using electronic densitometer (Alfa Mirage Co., Ltd. Model MD-300S0, Osaka, Japan) which employs Archimedes’ principle in order to calculate the density. Distilled water was used as the immersion fluid. The density was determined using the following equation [29]:

(1) $$\rho =\frac{W_{air}\times \rho_{dis.water}}{W_{air}-W_{dis.water}}$$

where $W_{dis.water}$ is the composite weight in distilled water, $W_{air}$ is the composite weight in air, and $\rho_{dis.water}$ is the density of the distilled water (1 gm/cm^3^).

#### 2.4.5. Dilatometer Linseis L75 Platinum

Dilatometer is one of the main techniques used for coefficients of thermal expansion (CTEs) measurements. In general, to determine the CTE, two physical quantities (temperature and displacement) must be measured on a specimen that is undergoing a thermal cycle. In this study, dilatometer (Linseis L75 Platinum, Selb, Germany) was used to determine the CTEs of composites. The sample was enclosed in the furnace and then heated up to 200 °C. When the sample expands, it pushes the central rod along the axis of the tube, and the relative movement can be characterized [30]. In addition, from an atomic perspective, the CTE represents the increment in the average distance among atoms with increasing temperature and, generally, weaker bonds have a higher CTE value [31].

#### 2.4.6. Extensometer (Shimadzu AGS-X 100kN)

rFe_2_O_3_–PTFE nanocomposites were fabricated as per the ASTM-D638 standard in order to find out the ultimate tensile strength (UTS) [32]. The influence of rFe_2_O_3_ nanoparticles on the UTS of PTFE matrix was examined using the Shimadzu (AGS-X 100kN, Kyoto, Japan) with industry-leading (Shimadzu, TRAPEZIUM X, Kyoto, Japan) data processing software. The pneumatic grips were displaced with a rate of 5 mm/min at room temperature. The data were obtained from the software and data acquisition system monitor continuously until the specimen reached its UTS and broke.

#### 2.4.7. Rectangular Waveguide (RWG)

**Figure 4:**
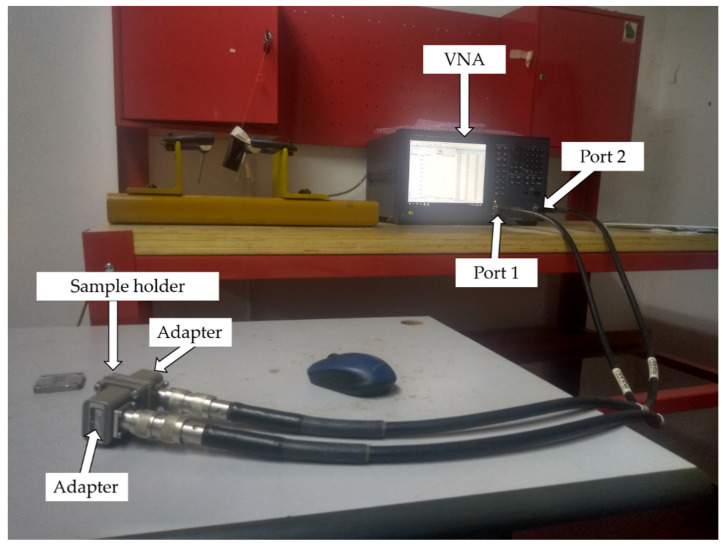
Measurement of complex permeability and permittivity using rectangular waveguide technique.

The measurements of relative complex permittivity and permeability were carried out in the frequency range of 8.2–12.4 GHz using rectangular waveguide (RWG) connected to a Keysight (E5063A) vector network analyzer (Keysight Technologies, Santa Rosa, CA, USA). As shown in Figure 4, the sample holder containing the sample was carefully attached to the flanges of RWG in order to totally avoid the air gap. The complex permittivity and permeability of the nanocomposites were deduced depending on the transmission and reflection of the waves throughout the composites. The measurement model of RWG to measure the permeability and permittivity was the poly-reflection–transmission µ and *ε* model. This model used an optimization technique which gives constant values for both $\varepsilon^{\prime }$ and µ′ throughout the whole frequency range. The transmission–reflection method is widely used in the EMI characterization because of the field-focusing ability which ensures an accurate characterization at the microwave frequency. The VNA was calibrated by implementing a standard full two-port calibration technique for 201 frequency points in the frequency range 8.2 GHz to 12.4 at room temperature. The characterization procedures were also described in our previous study [33].

## 3. Results and Discussion

### 3.1. Microstructural Characterization

**Figure 5:**
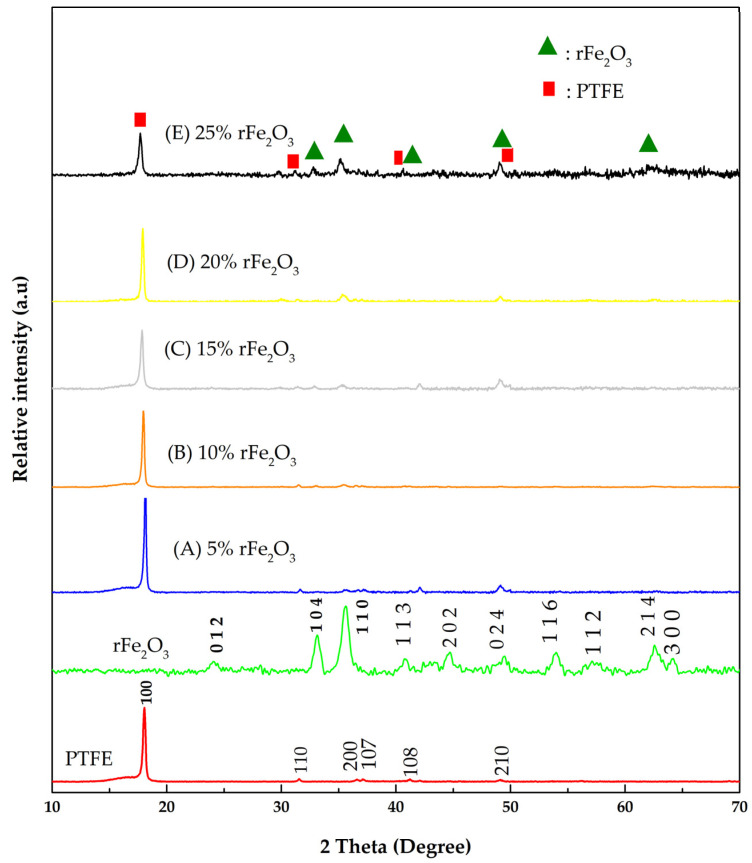
XRD diffractograms of the rFe_2_O_3_ nanoparticles, PTFE, and rFe_2_O_3_–PTFE nanocomposites.

The X-ray diffraction (XRD) technique was used to confirm the phase structurers of PTFE matrix, rFe_2_O_3_ nanopowder, and rFe_2_O_3_–PTFE nanocomposites. The XRD results are shown in Figure 5. By comparing the obtained diffractograms with the standard patterns from the Inorganic Crystal Structure Database (ICSD), the Bragg peaks of obtained recycled powder were all identified as rhombohedral crystal structure of single phase hematite (Fe_2_O_3_, Reference code: 00-024-0072) with R—3 space group. The diffractogram of rFe_2_O_3_ nanopowder showed the following peaks in the pattern at 2Ѳ of 24.1°, 33.1°, 35.6°, 40.8°, 43.5°, 49.4°, 54°, 57.5°, 62.4°, and 63.9° corresponding to the miller indices (hkl): (012), (104), (110), (113), (202), (024), (116), (122), (214), and (300), respectively. On the other hand, the diffractogram of PTFE matrix showed six peaks in the pattern at 2Ѳ of 18.04°, 31.54°, 36.60°, 37°, 41.22°, and 49.11°, which agreed with the study carried out by Yamaguchi et al. [34]. The mentioned peaks corresponded to the miller indices (hkl): (100), (110), (200), (107), (108), and (210), respectively. The diffractograms of rFe_2_O_3_–PTFE nanocomposites show dominant crystalline phase characteristics of PTFE at low concentration of rFe_2_O_3_ content in the nanocomposites. However, the peaks corresponding to the rFe_2_O_3_ nanopowder increased in intensity as the content increased. All peaks shown in rFe_2_O_3_–PTFE profiles belongs to the materials used for the preparation of the composites. The patterns did not show new peaks suggesting the nanocomposites were pure and there was no conversion of PTFE or rFe_2_O_3_ nanopowder into other materials. The absence of any new peaks of the nanocomposites also suggests that the rFe_2_O_3_ nanoparticles did not chemically interact with the PTFE matrix and that the mixture was physical in nature.

**Figure 6:**
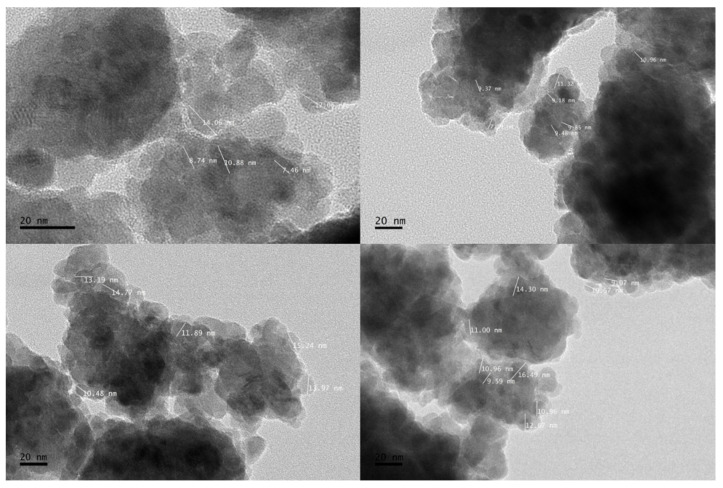
HRTEM images of rFe_2_O_3_ nanoparticles.

The microstructure characterization of the rFe_2_O_3_ nanoparticles was performed using high resolution transmission electron microscopy (HRTEM) to investigate the size and shape of rFe_2_O_3_ particles. The HRTEM results confirmed that the nanoparticles were highly crystalline as shown in Figure 6, which agreed with the study conducted by Wen et al. [35]. It can also be seen in Figure 6 that the rFe_2_O_3_ nanoparticles are aggregated and spherical, which may be attributed to the long-range magnetic dipole–dipole interaction between the particles. This observation may also be caused by the drying process during TEM sample preparation [36]. The rFe_2_O_3_ nanoparticles sizes were within the range 7.4–15.24 nm after 6 h of ball milling with an average particle size of 11.3 nm.

**Figure 7:**
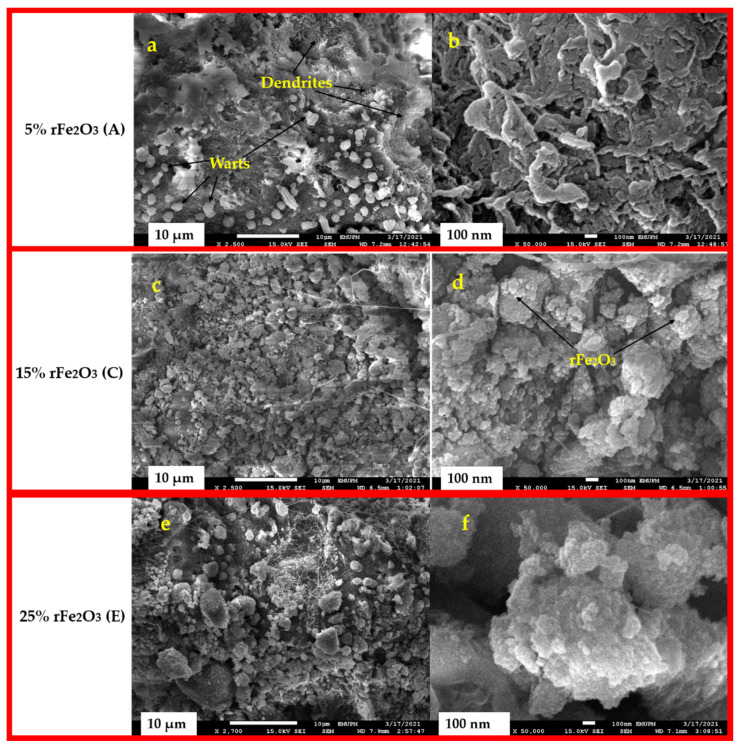
FESEM of rFe_2_O_3_–PTFE nanocomposites: 5% rFe_2_O_3_ (**A**) nanocomposite (**a**,**b**), 15% rFe_2_O_3_ (**C**) nanocomposite (**c**,**d**), and 25% rFe_2_O_3_ (**E**) nanocomposite (**e**,**f**).

The cross-sectional surface images of 5% rFe_2_O_3_ (A), 15% rFe_2_O_3_ (C), and 25% rFe_2_O_3_ (E) nanocomposite are shown in Figure 7. It can be seen in 5% nanocomposite (A) that there are two main types of patterns observed in PTFE structure. The first pattern is small outgrowths which are uniformly dispersed on the surface of PTFE and was called “warts”. The second pattern is “ribbon” like shapes which entirely cover the PTFE surface and was called “dendrites”. These observations are in accordance with the study conducted by Glaris et al. [37]. The images confirmed that with an increase in rFe_2_O_3_ loadings in the composites, the adhesion to PTFE matrix increased gradually. There were no agglomerates observed in rFe_2_O_3_–PTFE nanocomposites. The uniform dispersal of rFe_2_O_3_ nanoparticles in the PTFE matrix gives an indication that the nanoparticles were completely implanted in the nanocomposites and offered interfacial bonding which can enhance the complex permittivity and permeability.

### 3.2. Coefficients of Linear Thermal Expansion (CTEs)

**Figure 8:**
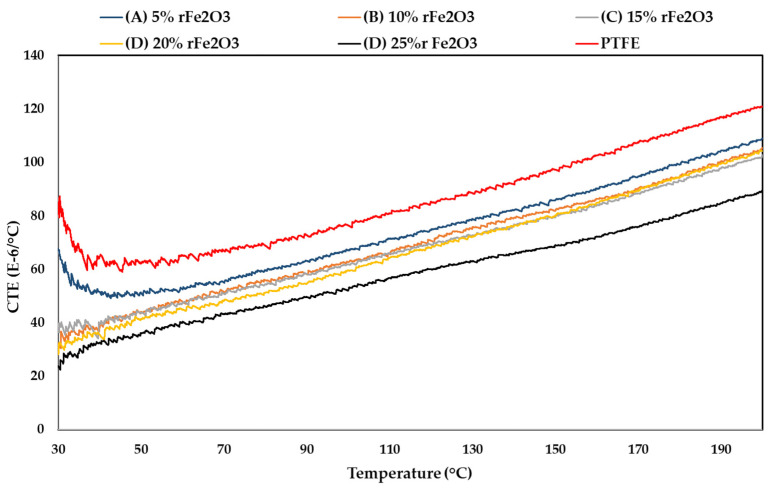
Thermal expansion coefficients of rFe_2_O_3_–PTFE nanocomposites.

The variation in CTEs with temperature for rFe_2_O_3_–PTFE nanocomposites in the temperature range 30–200 °C is presented in Figure 8. It was observed that the values of CTEs were low for the nanocomposites with higher content of rFe_2_O_3_ nanofiller. The decrease in CTEs of rFe_2_O_3_–PTFE nanocomposites is due to the difference between the CTEs of PTFE matrix and rFe_2_O_3_ nanofiller (Fe_2_O_3_ has a lower CTE comparing to PTFE). Figure 8 also shows that the CTEs of all nanocomposites increased as temperature increased throughout the measurement range. This behavior can be attributed to the increase in molecular vibrations with temperature, and a linear relationship exists between the CTE and the thermal capacitance per unit volume. The incorporation of low-CTE rFe_2_O_3_ in the PTFE matrix increased the thermal stability of the nanocomposites over a wide temperature range. Composites with low CTE are highly desirable for precision structures [38] such as microwave applications, where temperature fluctuations at the operating environment could cause substantial change in dimensions leading to destruction in the application.

### 3.3. Tensile Strength and Density of rFe_2_O_3_–PTFE Nanocomposites

**Figure 9:**
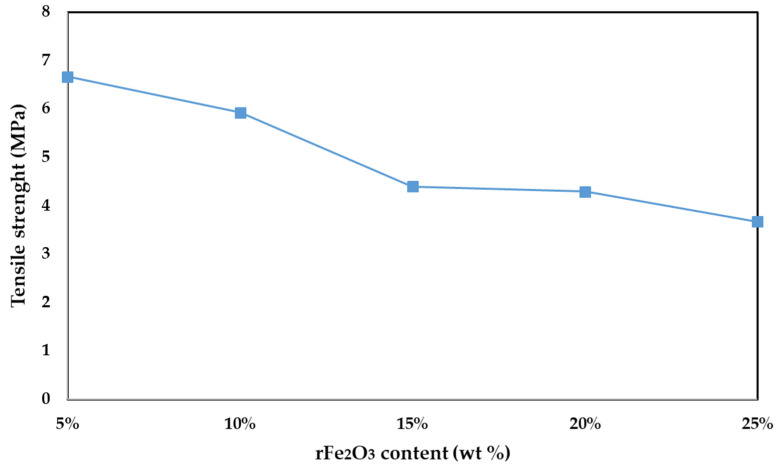
Tensile strength vs. various rFe_2_O_3_ nanofiller content for rFe_2_O_3_–PTFE nanocomposites.

The variation in tensile strength with filler content for different rFe_2_O_3_–PTFE nanocomposites is presented in Figure 9. The tensile strength of polymer composites depends on the properties of nanoparticle–matrix interaction which plays an important role in the level of dissipated energy by different damaging mechanisms which take place at the nanoscale [39]. As shown in Figure 9, the tensile strength of nanocomposites decreased with increasing rFe_2_O_3_ nanofiller content. This behavior was expectable for polymer composites with inorganic filler and it was reported by a study conducted by Jiang et al. [40]. This decrease could be attributed to the rFe_2_O_3_ nanofiller which were not able to support the stress transferred from PTFE matrix which weakened the nanocomposite [14].

**Figure 10:**
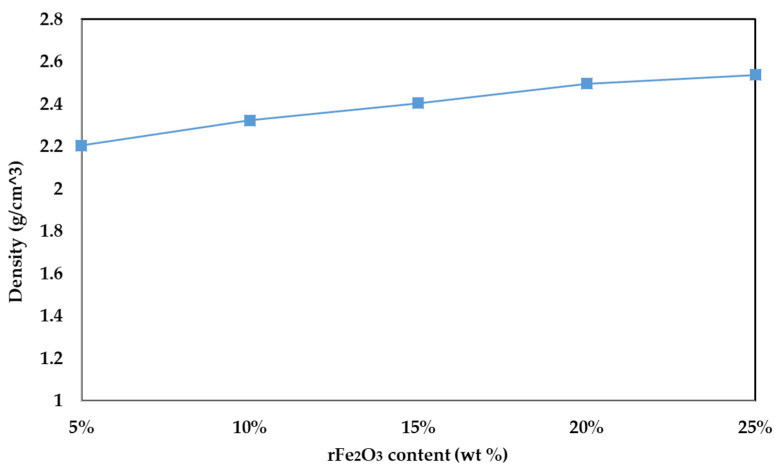
Density vs. various rFe_2_O_3_ nanofiller content for rFe_2_O_3_–PTFE nanocomposites.

The variation of density values with different rFe_2_O_3_ wt % content in the PTFE matrix is shown in Figure 10. The 5% (A), 10% (B), 15% (C), 20% (D), and 25% (E) rFe_2_O_3_ nanocomposites had the respective density values of 2.2, 2.32, 2.4, 2.49, 2.53 gm/cm^3^. Therefore, increasing the rFe_2_O_3_ nanofiller corresponded to increased density values of rFe_2_O_3_–PTFE nanocomposites. The increase in density with the increase in Fe_2_O_3_ nanofiller is supported by a study carried out by Ghasemi-Kahrizsangi et al. [41]. This behavior can be attributed to the higher true density of Fe_2_O_3_ (5.24 gm/cm^3^) [42], in comparison to the PTFE (2.2 gm/cm^3^) [43]. The increase in density can cause an observed increase in dielectric constant [44]. Moreover, Leyland and Maharaj found that the relationship between density and dielectric constant is directly proportional for various materials [45]. The denser rFe_2_O_3_–PTFE nanocomposites results higher number of molecules per unit volume. A larger number of molecules per unit volume means that there is more interaction with the electric fields and therefore an increase in the complex permittivity.

### 3.4. Complex Permittivity of rFe_2_O_3_–PTFE Nanocomposites

**Figure 11:**
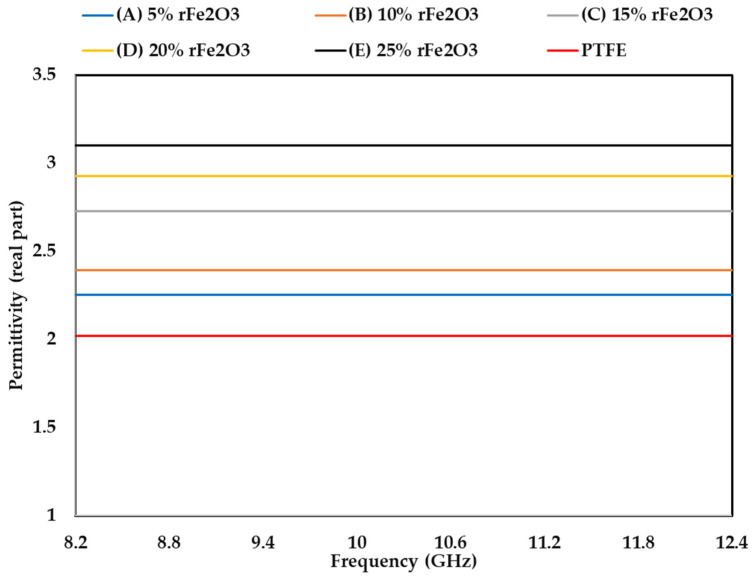
Variation in permittivity (real part) of rFe_2_O_3_–PTFE nanocomposites using rectangular waveguide.

**Figure 12:**
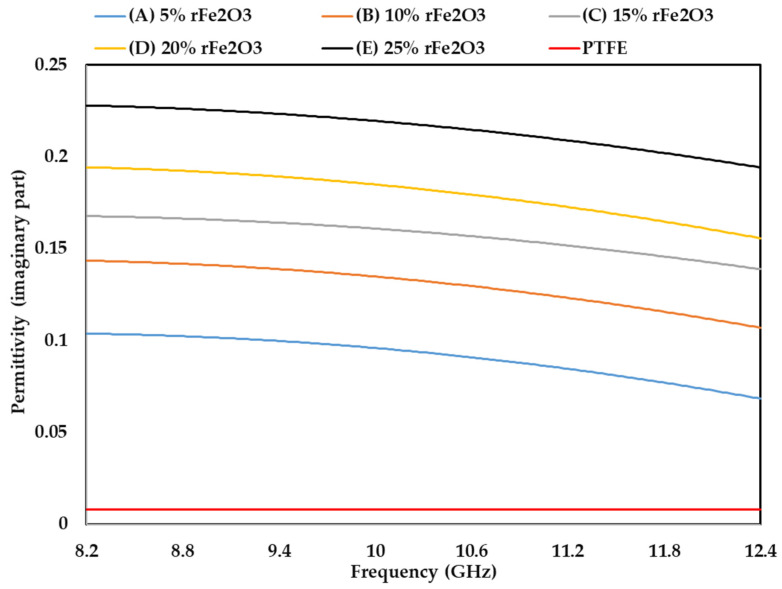
Variation in permittivity (imaginary part) of rFe_2_O_3_–PTFE nanocomposites using rectangular waveguide.

**Figure 13:**
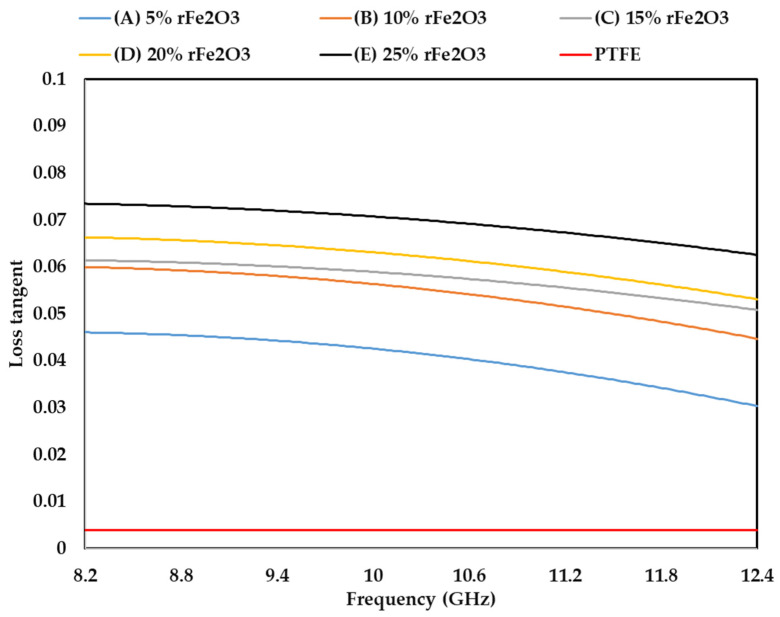
Variation in loss tangent of rFe_2_O_3_–PTFE nanocomposites.

The measured complex permittivity values (real and imaginary parts) of rFe_2_O_3_–PTFE nanocomposites in the frequency range of 8.2–12.4 GHz using rectangular waveguide are shown in Figure 11; Figure 12 while loss tangent is shown in Figure 13. The highest *ε*′ value of rFe_2_O_3_–PTFE nanocomposites was 3.1 for 25% rFe_2_O_3_ nanocomposite along the frequency range 8.2 GHz to 12.4 GHz while the lowest *ε*′ value was 2.01 for the PTFE sample. By increasing the rFe_2_O_3_ nanofiller in the nanocomposites, both *ε*′ and *ε*″ increased throughout the measurement range. It can be noticed that *ε*′ and *ε*″ increased from 2.29 and 0.10 for 5% rFe_2_O_3_ nanocomposite (A) to 2.39 and 0.14, respectively for 10% rFe_2_O_3_ nanocomposite (B) at 8.2 GHz. The same behavior of increasing complex permittivity by increasing the rFe_2_O_3_ nanofiller was observed in all nanocomposites. The *ε*′ and *ε*″ rely on the contribution of the atomic, electronic, interface, and orientation polarization in the sample [46]. The interface polarization increased due to the differences in polarization of the rFe_2_O_3_ nanofiller and PTFE matrix.

The increment in complex permittivity with increasing rFe_2_O_3_ nanofiller can be attributed to the polarization process due to the enhanced conductivity and interfacial polarization in the composite and hopping exchange of charges between localized states [23]. One more reason for the influence on the relative complex permittivity of the nanocomposites is the particle size of rFe_2_O_3_ nanofiller since the nano size had high specific surface area that enabled good contact between the particles and the matrix [47]. The increment in *ε*′ and *ε*″ at low frequency can be attributed to the dominant role of dipolar and interfacial polarization. However, the increment at high frequencies can be attributed to the electronic and ionic polarization of the system [48].

It can be also seen in Figure 12 that *ε*″ of rFe_2_O_3_–PTFE nanocomposites decreased by increasing the frequency from 8.2 to 12.4 GHz. This decrement can be attributed to the interfacial dipoles having less time to align in the direction of the external field. The molecules were able to do a complete orientation at low frequency but they were not able to achieve the same orientation at high frequency [49]. However, the *ε*″ of the PTFE sample did not change with frequency because PTFE is a nonpolar polymer [50], which means its complex permittivity is not dependent on frequency.

### 3.5. Complex Permeability of rFe_2_O_3_–PTFE Nanocomposites

**Figure 14:**
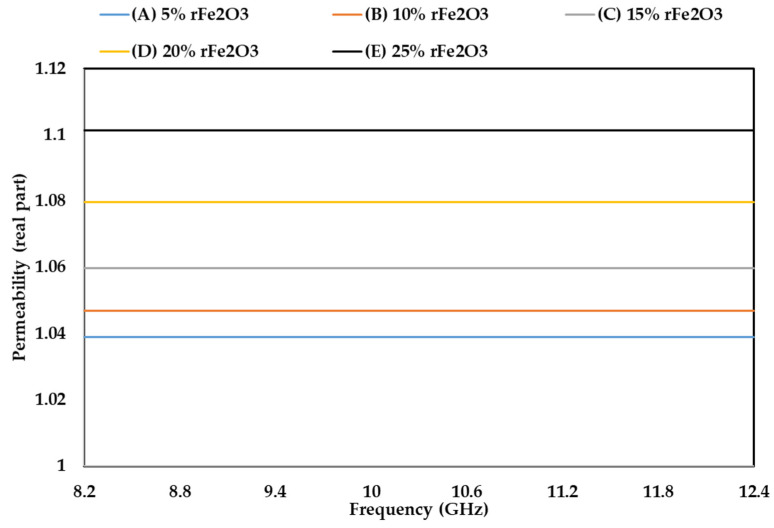
Variation in permeability (real part) of rFe_2_O_3_–PTFE nanocomposites using rectangular waveguide.

**Figure 15:**
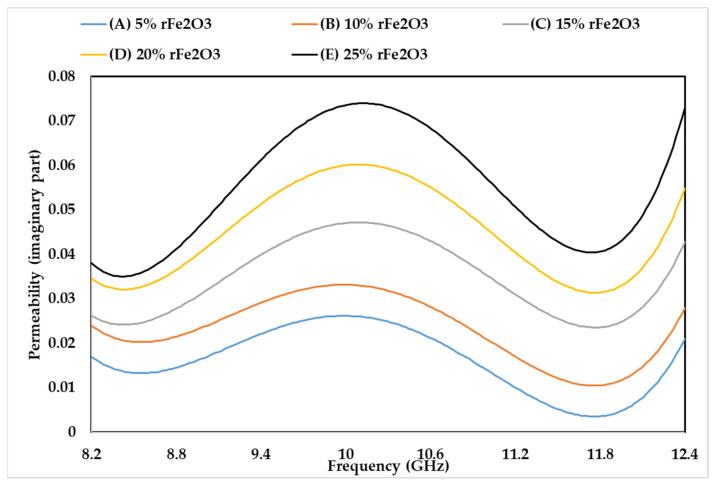
Variation in permeability (imaginary part) of rFe_2_O_3_–PTFE nanocomposites using rectangular waveguide.

**Figure 16:**
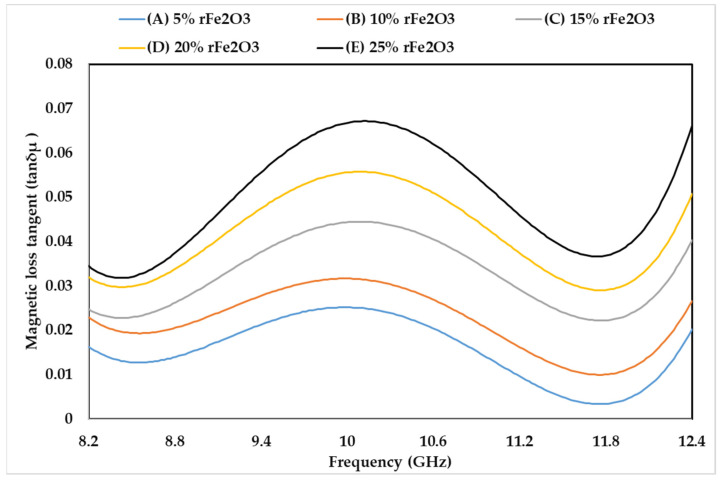
Variation in magnetic loss tangent of rFe_2_O_3_–PTFE nanocomposites.

The permeability characteristics of rFe_2_O_3_–PTFE nanocomposites can only be attributed to the rFe_2_O_3_ nanoparticles because PTFE is a non-magnetic material [51]. The variations in µ′, µ″, and $tan\delta_{µ}$ of rFe_2_O_3_–PTFE nanocomposites are shown in Figure 14, Figure 15 and Figure 16, respectively. It is clear that the values of µ′, µ″, and $tan\delta_{µ}$ were higher for rFe_2_O_3_–PTFE nanocomposites having higher rFe_2_O_3_ content.

As the content of rFe_2_O_3_ nanofiller increased in the rFe_2_O_3_–PTFE nanocomposites, the spacing in the nanocomposites reduced due to the high specific surface area of the rFe_2_O_3_ nanofiller. This made it more difficult for the magnetic field to pass through the rFe_2_O_3_–PTFE nanocomposites, thereby increasing the values of the relative complex permeability.

The real part (µ′) of the relative permeability values were 1.039, 1.047, 1.06, 1.8 and 1.1 for 5% (A), 10% (B), 15% (C), 20% (D) and 25% (E) rFe_2_O_3_ nanocomposites, respectively, within the frequency range of 8.2–12.4 GHz while the corresponding values of µ″ were 0.017, 0.023, 0.026, 0.034 and 0.038 at 8.2 GHz. A resonant peak can be clearly seen in each µ″ curve between 10 and 10.6 GHz. The increment in µ′ and µ″ of the polymer composites with increase in Fe_2_O_3_ is supported by studies conducted by Abdalhadi et al. [52] and Ahmad et al. [53]. In addition, the magnetic loss tangent in Figure 16 followed the behavior of µ″ because it was calculated using the equation $tan\delta_{µ}=\frac{{µ}^{\prime \prime }}{{µ}^{\prime }}$.

**Figure 17:**
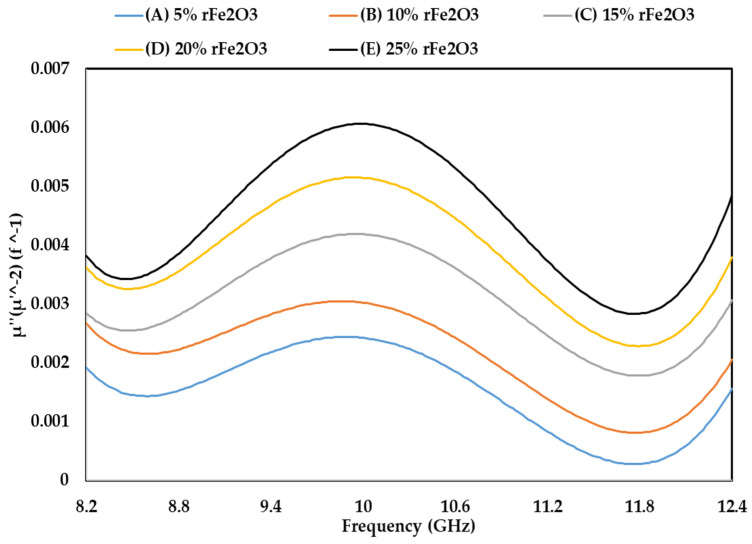
The values of ${µ}^{\prime }{\left({µ}^{\prime \prime }\right)}^{2}f^{-1}$ as a function of frequency for rFe_2_O_3_–PTFE nanocomposites.

It can be seen that µ′ in Figure 14 shows constant values while µ″ in Figure 15 shows non-steady behavior with the frequency. The µ′ is associated with the material storage capacity of the magnetic field [54], and it did not vary with frequency because of the RWG measurement model described in Section 2.4.7. On the other hand, the fluctuations in µ″ with the frequency can be attributed to the magnetic loss. In general, the magnetic loss of a sample originates because of one of these four reasons: the hysteresis loss, domain-wall resonance, eddy current effect, and natural resonance [55]. The first reason (hysteresis loss) which originates from the irreversible magnetization is negligible in a weak applied magnetization field [56]. The second reason (domain-wall resonance) normally occurs in the (1–100) MHz range, therefore, this reason can be excluded in this study [57]. The third reason (eddy current effect) can usually be estimated using the equation of $C_{0}={µ}^{\prime }{\left({µ}^{\prime \prime }\right)}^{2}f^{-1}$. When the magnetic loss originates for this reason, the $C_{0}\;$should be almost constant as the frequency varied. However, it can be seen in Figure 17 that the $C_{0}$ values for the rFe_2_O_3_–PTFE nanocomposites varied considerably with the frequency; so, this reason can also be excluded. Hence, these resonant peaks are associated with the natural ferromagnetic resonance [58]. The trend of µ″ is also supported by the study conducted by Liang [59].

## 4. Conclusions

In this research, rFe_2_O_3_–PTFE nanocomposites were successfully fabricated using rFe_2_O_3_ nanopowder as the filler and PTFE as the matrix. The effect of rFe_2_O_3_ loading on the structural, mechanical, thermal, dielectric, and magnetic properties of PTFE matrix was investigated. rFe_2_O_3_ nanofiller was dispersed uniformly with complete implantation in the PTFE matrix as presented in the FESEM results. The density increased but the tensile strength decreased with the increase of rF_2_O_3_ nanofiller in the nanocomposites. The rFe_2_O_3_ nanoparticles improved the thermal properties of the PTFE matrix. The complex permittivity and permeability were also enhanced by embedding rFe_2_O_3_ nanofiller in the matrix throughout the measurement range. The important dielectric, magnetic, thermal and mechanical properties of rFe_2_O_3_–PTFE nanocomposites linked to the content of the rFe_2_O_3_ in the nanocomposites can be employed in possible applications requiring tunable characteristics.

## Figures and Tables

**Table 1:** Composition of rFe_2_O_3_–PTFE nanocomposites.

Composite Code	Recycled Fe_2_O_3_ Nano-Powder		PTFE		Total Mass (g)
	Percentage %	Mass (g)	Percentage %	Mass (g)	
Control	0	0	100	60	60
A	5	3	95	57	60
B	10	6	90	54	60
C	15	9	85	51	60
D	20	12	80	48	60
E	25	15	75	45	60

## Data Availability

Not applicable.

## References

[1] Liu B., Zhang L., Zhang Y., Han G., Zhang B. (2021). Innovative methodology for co-treatment of mill scale scrap and manganese ore via oxidization roasting-magnetic separation for preparation of ferrite materials. Ceram. Int..

[2] Mensah E.E., Abbas Z., Azis R.S., Ibrahim N.A., Khamis A.M. (2019). Complex Permittivity and Microwave Absorption Properties of OPEFB Fiber–Polycaprolactone Composites Filled with Recycled Hematite (α-Fe_2_O_3_) Nanoparticles. Polymers.

[3] Eid C., Brioude A., Salles V., Plenet J.-C., Asmar R., Monteil Y., Khoury R., Khoury A., Miele P. (2010). Iron-based 1D nanostructures by electrospinning process. Nanotechnology.

[4] El-Leathy A., Danish S.N., Al-Ansary H., Jeter S., Al-Suhaibani Z. Experimental study of compatibility of reduced metal oxides with thermal energy storage lining materials. Proceedings of the AIP Conference Proceedings.

[5] Müller M., Villalba J.C., Mariani F.Q., Dalpasquale M., Lemos M.Z., Huila M.F.G., Anaissi F.J. (2015). Synthesis and characterization of iron oxide pigments through the method of the forced hydrolysis of inorganic salts. Dye. Pigment..

[6] Shi X., Zhou W., Ma D., Ma Q., Bridges D., Ma Y., Hu A. (2015). Electrospinning of nanofibers and their applications for energy devices. J. Nanomater..

[7] Bai H., Yin P., Lu X., Zhang L., Wu W., Feng X., Wang J., Dai J. (2020). Recent advances of magnetism-based microwave absorbing composites: An insight from perspective of typical morphologies. J. Mater. Sci. Mater. Electron..

[8] Raveendran A., Sebastian M.T., Raman S. (2019). Applications of microwave materials: A review. J. Electron. Mater..

[9] Bhattacharya P., Das C.K. (2013). In situ synthesis and characterization of CuFe_10_Al_2_O_19_/MWCNT nanocomposites for supercapacitor and microwave-absorbing applications. Ind. Eng. Chem. Res..

[10] Oladele I.O., Omotosho T.F., Adediran A.A. (2020). Polymer-Based Composites: An Indispensable Material for Present and Future Applications. Int. J. Polym. Sci..

[11] Chandra R.J., Shivamurthy B., Kulkarni S.D., Kumar M.S. (2019). Hybrid polymer composites for EMI shielding application—A review. Mater. Res. Express.

[12] Wu K.-T., Yuan Y., Zhang S.-R., Yan X.-Y., Cui Y.-R. (2013). ZrTi_2_O_6_ filled PTFE composites for microwave substrate applications. J. Polym. Res..

[13] Murali K., Rajesh S., Prakash O., Kulkarni A., Ratheesh R. (2009). Preparation and properties of silica filled PTFE flexible laminates for microwave circuit applications. Compos. Part A Appl. Sci. Manuf..

[14] Chen Y.-C., Lin H.-C., Lee Y.-D. (2003). The effects of filler content and size on the properties of PTFE/SiO_2_ composites. J. Polym. Res..

[15] Xie C., Liang F., Ma M., Chen X., Lu W., Jia Y. (2017). Microstructure and dielectric properties of PTFE-based composites filled by micron/submicron-blended CCTO. Crystals.

[16] Madusanka H., Samarasekara P., Fernando C. (2018). Polytetrafluoroethylene bindered hematite (α—Fe_2_O_3_) nanostructure for ammonia gas detection at room temperature. Mater. Res. Express.

[17] Kang W., Li F., Zhao Y., Qiao C., Ju J., Cheng B. (2016). Fabrication of porous Fe_2_O_3_/PTFE nanofiber membranes and their application as a catalyst for dye degradation. RSC Adv..

[18] Huang J.-Y., Fang X., Li Y.-C., Feng B., Wang H.-X., Du K. (2017). The mechanical and reaction behavior of PTFE/Al/Fe_2_O_3_ under impact and quasi-static compression. Adv. Mater. Sci. Eng..

[19] Ai Z., Mei T., Liu J., Li J., Jia F., Zhang L., Qiu J. (2007). Fe@ Fe_2_O_3_ core-shell nanowires as an iron reagent. 3. Their combination with CNTs as an effective oxygen-fed gas diffusion electrode in a neutral electro-Fenton system. J. Phys. Chem. C.

[20] Singh K., Ohlan A., Kotnala R., Bakhshi A., Dhawan S. (2008). Dielectric and magnetic properties of conducting ferromagnetic composite of polyaniline with γ-Fe_2_O_3_ nanoparticles. Mater. Chem. Phys..

[21] Reda S. (2013). electric and dielectric properties of Fe_2_O_3_/silica nanocomposites. Int. J. Nano Sci. Technol..

[22] Ul-Haq Y., Murtaza I., Mazhar S., Ullah R., Iqbal M., Qarni A.A., Amin S. (2020). Dielectric, thermal and mechanical properties of hybrid PMMA/RGO/Fe_2_O_3_ nanocomposites fabricated by in-situ polymerization. Ceram. Int..

[23] Mensah E.E., Abbas Z., Ibrahim N.A., Khamis A.M., Abdalhadi D.M. (2020). Complex permittivity and power loss characteristics of α-Fe_2_O_3_/polycaprolactone (PCL) nanocomposites: Effect of recycled α-Fe_2_O_3_ nanofiller. Heliyon.

[24] Paula A.L.D., Rezende M.C., Barroso J.J. (2011). Experimental measurements and numerical simulation of permittivity and permeability of Teflon in X band. J. Aerosp. Technol. Manag..

[25] Mensah E.E., Abbas Z., Azis R.a.S., Khamis A.M. (2019). Enhancement of complex permittivity and attenuation properties of recycled hematite (α-Fe_2_O_3_) using nanoparticles prepared via ball milling technique. Materials.

[26] Yusoff A., Abdullah M. (2004). Microwave electromagnetic and absorption properties of some LiZn ferrites. J. Magn. Magn. Mater..

[27] Wang Y., Wang L., Wu H. (2013). Enhanced microwave absorption properties of α-Fe_2_O_3_-filled ordered mesoporous carbon nanorods. Materials.

[28] Hong Y.-K., Lee J. (2013). Ferrites for RF passive devices. Solid State Phys..

[29] Ismail N.Q.A., Saat N.K., Zaid M.H.M. (2020). Effect of soda lime silica glass doping on ZnO varistor ceramics: Dry milling method. J. Asian Ceram. Soc..

[30] James J., Spittle J., Brown S., Evans R. (2001). A review of measurement techniques for the thermal expansion coefficient of metals and alloys at elevated temperatures. Meas. Sci. Technol..

[31] Wool R. (2005). Bio-Based Composites from Soybean Oil and Chicken Feathers.

[32] Shabana R., Sarojini J., Vikram K.A., Lakshmi V. (2019). Evaluating the mechanical properties of commonly used 3D printed ABS and PLA polymers with multi layered polymers. Int. J. Eng. Adv. Technol..

[33] Khamis A., Abbas Z., Ahmad A., Azis R.S., Abdalhadi D., Mensah E.E. (2020). Experimental and Computational Study on Epoxy Resin Reinforced with Micro-Sized OPEFB Using Rectangular Waveguide and Finite Element Method. IET Microw. Antennas Propag..

[34] Yamaguchi A., Kido H., Ukita Y., Kishihara M., Utsumi Y. (2016). Anisotropic pyrochemical microetching of poly (tetrafluoroethylene) initiated by synchrotron radiation-induced scission of molecule bonds. Appl. Phys. Lett..

[35] Wen B., Li J., Lin Y., Liu X., Fu J., Miao H., Zhang Q. (2011). A novel preparation method for γ-Fe_2_O_3_ nanoparticles and their characterization. Mater. Chem. Phys..

[36] Ang B., Yaacob I., Nurdin I. (2013). Investigation of Fe_2_O_3_/SiO_2_ nanocomposite by FESEM and TEM. J. Nanomater..

[37] Glaris P., Coulon J.-F., Dorget M., Poncin-Epaillard F. (2013). Thermal annealing as a new simple method for PTFE texturing. Polymer.

[38] Abusafieh A.A., Federico D.R., Connell S.J., Cohen E.J., Willis P.B. Dimensional stability of CFRP composites for space-based reflectors. Proceedings of the Optomechanical Design and Engineering 2001.

[39] Jahan M., Inakpenu R.O., Li K., Zhao G. (2019). Enhancing the mechanical strength for a microwave absorption composite based on graphene nanoplatelet/epoxy with carbon fibers. Open J. Compos. Mater..

[40] Jiang Z., Yuan Y. (2018). Effects of particle size distribution of silica on properties of PTFE/SiO2 composites. Mater. Res. Express.

[41] Ghasemi-Kahrizsangi S., Nemati A., Shahraki A., Farooghi M. (2016). Densification and properties of Fe_2_O_3_ nanoparticles added CaO refractories. Ceram. Int..

[42] Jedrzejewska A., Sibera D., Narkiewicz U., Pełech R., Jedrzejewski R. (2017). Effect of Synthesis Parameters of Graphene/Fe_2_O_3_ Nanocomposites on Their Structural and Electrical Conductivity Properties. Acta Phys. Pol. A.

[43] Gorshkov N., Goffman V., Vikulova M., Burmistrov I., Kovnev A., Gorokhovsky A. (2018). Dielectric properties of the polymer–matrix composites based on the system of Co-modified potassium titanate–polytetrafluorethylene. J. Compos. Mater..

[44] Lanza V., Herrmann D. (1958). The density dependence of the dielectric constant of polyethylene. J. Polym. Sci..

[45] Leyland R., Maharaj A. (2010). Dielectric constant as a means of assessing the properties of road construction materials. SATC.

[46] Fahad Ahmad A., Aziz S.H.A., Abbas Z., Mohammad Abdalhadi D., Khamis A.M., Aliyu U.S.A. (2020). Computational and Experimental Approaches for Determining Scattering Parameters of OPEFB/PLA Composites to Calculate the Absorption and Attenuation Values at Microwave Frequencies. Polymers.

[47] Przybyszewska M., Zaborski M. (2009). The effect of zinc oxide nanoparticle morphology on activity in crosslinking of carboxylated nitrile elastomer. Express Polym. Lett.

[48] Mandal S., Singh S., Dey P., Roy J., Mandal P., Nath T. (2016). Frequency and temperature dependence of dielectric and electrical properties of TFe2O4 (T=Ni, Zn, Zn0.5Ni0.5) ferrite nanocrystals. J. Alloys Compd..

[49] Ahmad A.F., Ab Aziz S., Abbas Z., Obaiys S.J., Khamis A.M., Hussain I.R., Zaid M.H.M. (2018). Preparation of a chemically reduced graphene oxide reinforced epoxy resin polymer as a composite for electromagnetic interference shielding and microwave-absorbing applications. Polymers.

[50] Giacometti J.A., Wisniewski C., Ribeiro P.A., Moura W.A. (2001). Electric measurements with constant current: A practical method for characterizing dielectric films. Rev. Sci. Instrum..

[51] Huashen W., Shan J., Guodong W., Ke X. Electromagnetic parameters test system based on a refined NRW transmission/reflection algorithm. Proceedings of the 2007 International Symposium on Microwave, Antenna, Propagation and EMC Technologies for Wireless Communications.

[52] Abdalhadi D.M., Abbas Z., Ahmad A.F., Matori K.A., Esa F. (2018). Controlling the properties of OPEFB/PLA polymer composite by using Fe_2_O_3_ for microwave applications. Fibers Polym..

[53] Ahmad A.F., Abbas Z., Obaiys S.J., Abdalhadi D.M. (2017). Improvement of dielectric, magnetic and thermal properties of OPEFB fibre–polycaprolactone composite by adding Ni–Zn ferrite. Polymers.

[54] Barba A., Clausell C., Jarque J., Nuño L. (2020). Magnetic complex permeability (imaginary part) dependence on the microstructure of a Cu-doped Ni–Zn-polycrystalline sintered ferrite. Ceram. Int..

[55] Luo J., Shen P., Yao W., Jiang C., Xu J. (2016). Synthesis, characterization, and microwave absorption properties of reduced graphene oxide/strontium ferrite/polyaniline nanocomposites. Nanoscale Res. Lett..

[56] Li W., Qiu T., Wang L., Ren S., Zhang J., He L., Li X. (2013). Preparation and electromagnetic properties of core/shell polystyrene@ polypyrrole@ nickel composite microspheres. ACS Appl. Mater. Interfaces.

[57] Liu P., Huang Y., Yan J., Zhao Y. (2016). Magnetic graphene@ PANI@ porous TiO_2_ ternary composites for high-performance electromagnetic wave absorption. J. Mater. Chem. C.

[58] Li C., Ji S., Jiang X., Waterhouse G.I., Zhang Z., Yu L. (2018). Microwave absorption by watermelon-like microspheres composed of γ-Fe_2_O_3_, microporous silica and polypyrrole. J. Mater. Sci..

[59] Liang K., Qiao X.-J., Sun Z.-G., Guo X.-D., Wei L., Qu Y. (2017). Preparation and microwave absorbing properties of graphene oxides/ferrite composites. Appl. Phys. A.

